# FN3K deficiency drives tubular injury in diabetic kidney disease through impaired mitochondrial respiratory chain function

**DOI:** 10.3389/fendo.2026.1901830

**Published:** 2026-08-24

**Authors:** Dehan Cai, Jing Wu, Xionghui Yang, Mingjie He

**Affiliations:** Department of Nephrology, Jiangxi Provincial People’s Hospital, The First Affiliated Hospital of Nanchang Medical College, Nanchang, Jiangxi, China

**Keywords:** diabetic kidney disease, fructosamine-3-kinase, mitochondrial homeostasis, PI3K/Akt signaling pathway, tubular injury

## Abstract

**Aims:**

Despite growing evidence implicating FN3K in the pathogenesis of cancer and diabetes, its precise role in modulating renal tubular injury within the context of diabetic nephropathy has yet to be fully elucidated.

**Materials and methods:**

FN3K expression was examined in DKD model mice, human kidney biopsy samples, and high glucose-treated HK-2 cells via immunohistochemistry (IHC) and Western blotting. Associations between serum FN3K levels and DKD risk were analyzed in the Kidney Precision Medicine Project cohort via multivariable logistic regression, restricted cubic spline modelling, and receiver operating characteristic (ROC) analysis. Regional proteomics, single-cell RNA sequencing, untargeted metabolomics, and joint pathway analyses were integrated to delineate FN3K-associated pathways and cell type-specific metabolic alterations. The effect of FN3K on mitochondrial function was evaluated using Mitochondrial respiratory chain activity assays.

**Results:**

FN3K expression was significantly reduced in the renal tubular compartments of DKD mice and patients and was downregulated by high glucose in HK-2 cells. Low circulating FN3K levels were independently associated with increased DKD risk and improved diagnostic performance when combined with conventional biomarkers. Proteomic and single-cell analyses revealed the activation of PI3K/AKT signaling and the suppression of mitochondrial metabolic pathways in DKD tubules, whereas FN3K-high proximal tubule cells presented increased oxidative phosphorylation and reduced inflammatory and apoptotic signaling. Metabolomic profiling demonstrated that FN3K overexpression restored the levels of mitochondrial- and nitrogen-related metabolites and attenuated injury-associated lipid remodeling. Joint pathway analysis highlighted nitrogen and glutathione metabolism as convergent FN3K-related signatures. Over-expression of FN3K would improves the function of mitochondrial respiratory chain I and III activity.

**Conclusions:**

FN3K deficiency is associated with tubular injury and DKD progression, potentially through increased glycation stress, mitochondrial metabolic dysfunction.

## 1 Background

Diabetic kidney disease (DKD) is one of the most common chronic complications of diabetes and represents the leading cause of chronic kidney disease (CKD) worldwide (1). Persistent hyperglycemia contributes to renal injury through multiple mechanisms, including tubular epithelial cell damage, disruption of the glomerular filtration barrier, and activation of inflammatory responses (2). In parallel, sustained elevations in circulating glucose promote the formation of advanced glycation end products (AGEs) (3). Accumulating evidence indicates that increased AGE burden is closely associated with a progressive decline in renal function and adverse renal outcomes in patients with diabetes (4).

AGE formation occurs through a series of complex nonenzymatic reactions collectively referred to as the Maillard reaction (5, 6). This process is initiated by the interaction of reducing sugars, such as glucose or fructose, with proteins, nucleic acids, or lipids to form reversible Schiff base intermediates. These intermediates subsequently rearrange into more stable Amadori products, which undergo further oxidative and chemical modifications to generate irreversible AGEs (5). Current strategies aimed at mitigating AGE-related tissue injury largely focus either on limiting AGE formation at early stages or on attenuating the downstream biological effects of AGEs (6). However, the mechanisms underlying excessive AGE accumulation in DKD, particularly those related to impaired endogenous glycation repair, remain incompletely understood.

Fructosamine-3-kinase (FN3K), first identified in 2001, functions as a key degradation enzyme by phosphorylating early glycation adducts, thereby facilitating their removal and limiting their progression toward irreversible AGE formation (7, 8). FN3K activity is enriched in organs highly susceptible to nonenzymatic glycation, including the brain, heart, kidney, and skeletal muscle, suggesting a protective role against hyperglycemia-induced injury (9, 10). Deficiency of FN3K has been associated with increased levels of glycated hemoglobin, indicating impaired glycation repair capacity (11). Moreover, multiple studies have reported protective roles for FN3K in both microvascular and macrovascular complications of diabetes (12–19). Experimental evidence further suggests that FN3K modulation may exert beneficial effects on conditions such as cataract formation, age-related macular degeneration, and diabetic skin damage (20, 21). In contrast, inhibition of FN3K has been reported to increase drug sensitivity in certain cancers, underscoring its context-dependent biological functions (22). Collectively, these findings highlight FN3K as a critical regulator of glycation stress, yet its expression pattern and mechanistic role in DKD remain poorly defined.

Renal tubular injury is increasing recognized as an early and a primary event in DKD that can occur independently of or even precede overt glomerular damage (23). Notably, proximal tubular epithelial cells have high energy demands to sustain solute reabsorption, they rely heavily on mitochondrial oxidative phosphorylation and fatty acid β-oxidation, rendering them intrinsically vulnerable to metabolic stress in the diabetic milieu. Consequently, mitochondrial dysfunction as a central driver of tubular injury in DKD (24, 25). Reduced tubular oxidative metabolism and tricarboxylic acid cycle disruption prior to the onset of albuminuria such derangements are observed in kidney biopsies from young patients with diabetes (26). Importantly, AGEs appear to directly contribute to this process, as AGE accumulation in proximal tubular cells has been shown to trigger RAGE-dependent oxidative stress that impairs mitochondrial membrane integrity and ATP generation, linking glycation stress to tubular mitochondrial injury (27). Despite this converging evidence, the endogenous mechanisms that regulate AGE-induced mitochondrial damage in renal tubular cells remain largely unexplored.

In the present study, we investigated the expression and potential pathogenic role of FN3K in diabetic kidney disease. FN3K expression was first assessed in kidney tissues from patients with DKD, DKD model mice, and DKD-relevant cellular models. We then analyzed the associations between circulating FN3K levels and DKD-related clinical indices. Multi-omics approaches-integrating transcriptomics, proteomics, metabolomics, and lipidomics-have emerged as powerful strategies for dissecting the molecular mechanisms underlying kidney disease and for identifying novel diagnostic and therapeutic targets (28–33). Consequently, integrative analyses of proteomic profiling and single-cell RNA sequencing (scRNA-seq) datasets were performed to explore the molecular pathways and cell type-specific patterns linking FN3K to DKD progression. Finally, we validated the potential mechanism of FN3K through metabolomics and experimental methods.

## 2 Methods

### 2.1 Cell culture and materials

Human renal proximal tubular epithelial cells (HK-2, ATCC, USA) were maintained in minimum essential medium (MEM) (Procell System, China) supplemented with 10% fetal bovine serum (Gibco, USA) and 1% penicillin-streptomycin (MedChemExpress, USA) at 37 °C in a humidified atmosphere containing 5% CO_2_. After resuscitation from cryopreservation, cells were allowed to recover for 48h and confirmed to have normal morphology before use in subsequent experiments. To mimic DKD-related injury, cells were treated with high glucose (HG; MedChemExpress, USA) (45 and 65 mmol/L) for the indicated durations, based on the concentrations mentioned in previous literature (34). Where indicated, an osmotic control was included with mannitol (65 mmol/L, MedChemExpress, USA).

### 2.2 FN3K overexpression in HK-2 cells via plasmid transfection

FN3K overexpression in HK-2 cells was achieved via transient plasmid transfection. Briefly, HK-2 cells were seeded in culture plates and grown to approximately 60-80% confluence. The FN3K overexpression plasmid was diluted in serum-free Opti-MEM, mixed with a suitable transfection reagent (Lipo8000, Beyotime, China) according to the manufacturer’s instructions, and then incubated at room temperature to allow complex formation. The DNA-reagent complexes were added dropwise to the cells, which were subsequently incubated under standard culture conditions. After 4 h, the medium was replaced with fresh complete medium, and the cells were further cultured for 48 h under HG conditions. The overexpression efficiency was confirmed by Western blotting.

### 2.3 DKD mouse animal models

Male C57BL/6J mice (6–8 weeks old), purchased from Shulaibao (Wuhan) Biotechnology Co., Ltd., were housed under a controlled temperature and a 12-hour light/dark cycle with free access to food and water. Then, were fed a high-fat diet (60% kcal from fat [HKeyBio, cat: SYHF60]) for 8 weeks. After 8 weeks of high-fat feeding, mice were fasted for 12 h and administered a single intraperitoneal injection of streptozotocin (STZ, 100 mg/kg, freshly dissolved in 0.1 M citrate buffer, pH 4.5). Diabetes was confirmed 7 days after STZ injection when fasting blood glucose exceeded 11 mmol/L. Renal injury was evaluated via serum creatinine level and histology. Kidney tissues were harvested for IHC.

All animal procedures were approved by the Animal Ethics Committee of the First Affiliated Hospital of Nanchang Medical College and conducted in accordance with relevant guidelines.

### 2.4 Patients with DKD enrollment

Patients with DKD were recruited from Jiangxi Provincial People’s Hospital in 2025, and healthy kidney biopsy samples were obtained from kidney stone donors. DKD was defined according to guideline-based clinical criteria. The exclusion criteria included nondiabetic kidney disease, acute infection, active malignancy, autoimmune disease, and pregnancy. Demographic information and clinical parameters, including age, sex, diabetes duration, HbA1c, eGFR, albuminuria/proteinuria, blood pressure, and lipid profile, were collected. Kidney samples were obtained from biopsies for histological validation. The study protocol was approved by the ethics committee of the Ethics Committee of Jiangxi Provincial People’s Hospital.

### 2.5 Single-cell RNA sequencing analysis

Single-cell transcriptomic data extracted from the Kidney Precision Medicine Project (KPMP) (ID: 521c5b34-3dd0-4871-8064-61d3e3f1775a_PREMIERE_Alldatasets_08132021.h5Seurat) were processed via Seurat (version 5.4.0). Low-quality cells were excluded on the basis of predefined thresholds for gene counts, UMI counts, and mitochondrial transcript proportions. After normalization and scaling, dimensionality reduction was performed via principal component analysis (PCA) followed by clustering and visualization via UMAP. Cell identities were assigned via canonical marker genes and validated with established reference annotations where relevant. The genes with higher expression and lower expression in the proximal tubules (PTs) of the FN3K group were subsequently grouped according to the median FN3K expression. Finally, enriched pathways were evaluated via KEGG and enrichment analyses.

### 2.6 Regional proteomic analysis

We filtered 5 patients with DKD and 5 controls with normal kidney functions and extracted regional proteomic raw LC-MS/MS data from the KPMP. The raw mass spectrometry data were subsequently retrieved via Proteomo Discoverer (v2.4). The data type is off-label quantitative proteomics data based on primary parent ions. The database was sourced from the UniProt database Homo_sapiens_9606_proteome_20240830 (release: 2024-08-30, sequence: 204411), and common contaminant libraries were added to the database. Contracting proteins were removed during data analysis. The digestion method was set to trypsin/P. The number of missed cut-off sites was set to 2. The first search and main search were set to 20 ppm and 5 ppm for the parent ion and 20 ppm for the secondary fragment ions, respectively. The fixed modification was cysteine alkylation, and the variable modifications were oxidation of methionine and acetylation of the protein N-terminus. The FDRs for protein identification and PSM identification were set to 1%. Differentially expressed proteins were determined using the following thresholds: |log2-fold change| > 1.5 and adjusted P value <0.05. We annotated protein pathways via the Kyoto Encyclopedia of Genes and Genomes (KEGG) pathway database. The identified proteins were subjected to BLAST alignment (BLASTP, E value ≤ 1e−4). For each sequence, the BLAST hit with the highest alignment score was selected for annotation. We used the subcellular localization prediction software WoLF PSORT to annotate the subcellular localization of the submitted proteins. Protein domains were annotated for the identified proteins via the Pfam database and the corresponding PfamScan tool. The Pfam database was used to assess the enrichment of functional domains among the differentially expressed proteins. Fisher’s exact test was applied to evaluate the significance of domain enrichment in the differentially expressed proteins, using all identified proteins as the background. A P value < 0.05 was considered statistically significant.

### 2.7 Untargeted metabolomics analysis

To explore the effect of FN3K on metabolism, we established a FN3K-overexpressing cell model and divided the cells into two groups: high glucose treatment (n=3) and high plus overexpression of FN3K (n=3). Metabolites from the cell samples were extracted via cold methanol-based protocols followed by protein precipitation at -20 °C and high-speed centrifugation; the resulting supernatants were collected for UPLC–HRMS analysis, and pooled quality control (QC) samples were prepared by mixing equal aliquots of all the sample supernatants. Metabolomic profiling was performed on UPLC systems coupled to high-resolution mass spectrometers (TripleTOF 6600, Q Exactive Plus, or Orbitrap Exploris 120) operated in both positive and negative electrospray ionization modes via an ACQUITY UPLC HSS T3 column with acetonitrile–aqueous ammonium acetate/acetic acid mobile phases and gradient elution. Raw LC-MS data were converted to mzXML data and processed via XCMS/CAMERA/metaX in R for peak detection, alignment, grouping, and annotation of isotopes/adducts, generating an ion feature matrix (RT–m/z, samples, intensities). Metabolite annotation was conducted by matching accurate masses (≤10 ppm) to KEGG and HMDB, with molecular formula confirmation aided by isotope patterns and an in-house MS/MS spectral library. Statistical analyses were performed in R, including data filtering, KNN-based missing value imputation, and probabilistic quotient normalization (PQN), followed by multivariate analysis (PCA), correlation analysis (Pearson), and differentially abundant metabolite identification via t tests (P < 0.05, fold change > 1.2). KEGG pathway enrichment was assessed by hypergeometric testing (P < 0.05), metabolite set enrichment analysis was performed via GSEA (|NES| > 1, nominal P < 0.05, FDR < 0.25), and pathway-based network diagrams were constructed to visualize metabolite interactions.

### 2.8 Joint pathway analysis

We performed a joint pathway analysis in MetaboAnalyst to integrate metabolomics data with scRNA-seq data. Briefly, proximal tubule (PT) cells were extracted from the scRNA-seq dataset, and the samples/cells were stratified into the FN3K-high and FN3K-low groups on the basis of their FN3K expression; the genes differentially expressed between these two groups were used as the transcriptomic input for integration. In parallel, differentially abundant metabolites were identified from the metabolomics dataset. The resulting gene list and metabolite list (with corresponding identifiers mapped to KEGG) were uploaded to MetaboAnalyst’s Joint Pathway Analysis module, where features were matched to shared KEGG pathways and evaluated via combined enrichment/topology-based statistics to highlight pathways that were consistently perturbed at both the gene and metabolite levels. The significantly enriched joint pathways were then summarized and visualized to interpret coordinated metabolic and transcriptional alterations associated with FN3K expression in PT cells.

### 2.9 Correlations between serum FN3K levels and clinical indices

We extracted serum FN3K expression and clinical indices from the KPMP dataset. The non-DKD (NDKD) group, which included 46 people, was filtered by normal kidney function (estimated glomerular filtration barrier >90 ml/min/1.^73 m2)^, and the DKD group, which included 84 patients, was filtered by primary adjudicated category, as shown in **Table 1**. Continuous variables were assessed for normality via the Shapiro–Wilk test and are presented as the means ± standard deviations (SDs) or medians (interquartile ranges, IQRs) as appropriate. Categorical variables were compared via Fisher’s exact test. The associations between serum FN3K levels (FN3K expression levels were scaled), clinical parameters and DKD were subsequently evaluated via single-logistic analysis. The significant indices were subsequently added to the multivariable logistic models. We also divided the patients into three groups, T1, T2, and T3, on the basis of the expression of FN3K to explore the relationships between the FN3K groups and DKD risk via logistic analysis. To further identify the nonlinear association between FN3K and DKD, we performed multivariable logistic regression via the rms package to evaluate the association between FN3K expression and DKD while adjusting for age, sex, race, albumin, HbA1c category, cystatin C, and blood urea nitrogen. FN3K expression was modelled with a restricted cubic spline (4 knots) to allow for potential nonlinear effects. We then used an ANOVA function to test both the overall association and the nonlinearity of the spline term and generated adjusted predictions with the Predict function, exponentiating the linear predictor to obtain odds ratios (ORs) with 95% confidence intervals referenced to an OR of 1. To analyze the efficacy of FN3K expression and other clinical indices in the diagnosis of DKD, we constructed a receiver operating characteristic (ROC) curve.

**Table 1:** Baseline clinical characteristics of participants with nondiabetic kidney disease (NDKD) and diabetic kidney disease (DKD).

Clinical index	NDKD	DKD	P
Sex	Male: 20; Female: 26	Male: 48; Female: 34	1.01E-01
Race			7.36E-02
Asian	1	4	
Black or African-American	7	28	
White	34	43	
Other	4	7	
Age			<0.0001
20-29Years	3	0	
30-39Years	11	6	
40-49Years	13	12	
50-59Years	15	19	
60-69Years	4	35	
70-79Years	0	10	
eGFR (ml/min/1.73m^2^)	106.16 ± 10.43	49.12 ± 15.51	2.10E-49
FN3K (Scale)	0.30 ± 1.03	-0.17 ± 0.94	1.18E-02
Albumin (g/dL)	4.18 ± 0.54	3.86 ± 0.58	2.50E-03
Blood urea nitrogen (mg/dL)	17.41 ± 13.23	31.36 ± 11.42	5.17E-08
Creatinine (mg/dL)	1.39 ± 2.13	1.64 ± 0.55	4.39E-01
Cystatin C (mg/L)	0.69 ± 0.95	1.19 ± 1.02	7.48E-03
HbA1c (>=7%)	10	45	4.00E-04

The data are presented as the means ± standard deviations (SDs) for continuous variables and as counts for categorical variables. The estimated glomerular filtration rate (eGFR) is expressed as ml/min/1.73 m². The FN3K levels were standardized (scaled) prior to analysis. Comparisons between groups were performed via Student’s *t* test for continuous variables and Fisher’s exact test for categorical variables. P values indicate differences between the NDKD and DKD groups.

### 2.10 IHC analysis

Paraffin sections were deparaffinized in eco-friendly dewaxing solutions I–III (10 min each), rehydrated through three changes of absolute ethanol (5 min each), and rinsed in distilled water. Antigen retrieval was then performed as specified, ensuring that the sections remained wet throughout; after natural cooling, the slides were washed in PBS (pH 7.4) three times (5 min each). Endogenous peroxidase activity was blocked with 3% methanol-H_2_O_2_ for 25 min at room temperature in the dark, followed by washing with PBS. The sections were blocked with 3% BSA for 30 min at room temperature (rabbit serum was used when the primary antibody was goat derived), incubated with primary antibody (FN3K) diluted in PBS (1:100) overnight at 4 °C, and then with the corresponding HRP-conjugated secondary antibody for 50 min at room temperature after being washed with PBS. DAB was freshly prepared and applied for color development under microscopic monitoring, and the reaction was stopped with running water. Nuclei were counterstained with hematoxylin (~3 min), differentiated, blued, and washed, followed by dehydration through graded alcohols, clearing (n-butanol and xylene), and mounting with mounting medium. Finally, the slides were evaluated under a bright-field microscope. The H score was quantified via Saiviewer with consistent thresholding (H-Score(∑ (pi× i)=(percentage of weak intensity cells×1)+(percentage of moderate intensity cells×2)+(percentage of strong intensity cells×3).

### 2.11 Western blotting analysis

The cells were lysed in RIPA buffer supplemented with protease and phosphatase inhibitors, incubated on ice, and centrifuged at 4 °C to collect the protein supernatant. Proteins were mixed with SDS loading buffer, denatured, separated by SDS-PAGE and transferred onto PVDF membranes. The membranes were blocked with 5% nonfat milk or 5% BSA in TBST and incubated with primary antibodies, FN3K (Proteintech, Cat No. 14293-1-AP, dilution: 1:1000) and tubulin (Servicebio, Cat No. GB15140-100, dilution: 1:1000) at 4 °C overnight. After being washed with TBST, the membranes were incubated with appropriate HRP-conjugated secondary antibodies (diluted 1:10000) at room temperature for 2 hours, washed again, and visualized via enhanced chemiluminescence (ECL). Band intensities were quantified by Image Lab and normalized to those of tubulin.

### 2.12 Detection of mitochondrial respiratory chain complex I and III activity

The cells were collected from six-well plates and resuspended in 1.0 mL of extraction buffer, followed by homogenization on ice. The homogenate was centrifuged at 600 × g for 10 min at 4 °C, and the supernatant was further centrifuged at 11,000 × g for 15 min at 4 °C. The supernatant was discarded, and the pellet was used for measurement of mitochondrial complex activity. For Complex I, the pellet was resuspended in 200 μL of Extraction Buffer I and 200 μL of Extraction Buffer II; for Complex III, the pellet was resuspended in 200 μL of extraction buffer. Both were subjected to ultrasonic disruption at 200 W (5s on, 10s off, 15 cycles). Complex I (NADH-CoQ reductase) activity was measured by monitoring the oxidation rate of NADH at 340 nm via a UV spectrophotometer. The reaction was carried out at 37 °C for 1 min, and the absorbance was recorded at 10 s (A1) and 1 min 10 s (A2). ΔA = A1 − A2. The enzyme activity was calculated as follows: complex I activity (U/mg prot) = 3215.43 × ΔA ÷ Cpr. Complex III (CoQ-cytochrome c reductase) activity was measured by monitoring the rate of reduced cytochrome c formation at 550 nm via a visible spectrophotometer. The absorbance was recorded at 10 s (A1) and 2 min (A2). A blank control was included for each assay tube, in which Reagent 3 was replaced with distilled water. ΔA = (A2sample − A1sample) − (A2blank − A1blank). Enzyme activity was calculated as follows: complex III activity (U/mg prot) = 262 × ΔA ÷ Cpr.

For both complexes, one unit of enzyme activity was defined as the amount of enzyme that catalyzes the consumption or production of 1 nmol substrate per mg protein per minute. The protein concentration was determined with correction for the protein content of the extraction buffer itself. All assay kits were purchased from Beijing Solarbio Science & Technology Co., Ltd. (Complex I, cat. no. BC0515; Complex III, cat. no. BC3245).

### 2.13 Assessment of mitochondrial morphology

Following the indicated treatments, HK-2 cells were incubated with MitoTracker Red CMXRos (Servicebio, Cat No. G1723-50UG) at a final concentration of 400 nM in pre-warmed serum-free medium for 30 min at 37 °C in the dark. Cells were then washed three times with pre-warmed PBS. Fluorescence images were acquired using a confocal microscope.

### 2.14 Statistical analysis

Analyses were performed via R (version 4.5.2) and GraphPad Prism (version 10.1.2). Continuous variables are presented as the means ± SDs as appropriate. Between-group comparisons were conducted via Student’s t test, and multiple-group comparisons were performed via ANOVA and the Kruskal-Wallis test. For multiomics analyses, P values were adjusted via the Benjamini–Hochberg procedure. A two-sided P < 0.05 was considered statistically significant.

## 3 Results

### 3.1 Reduced FN3K expression in renal tubular compartments in DKD

**Figure 1:**
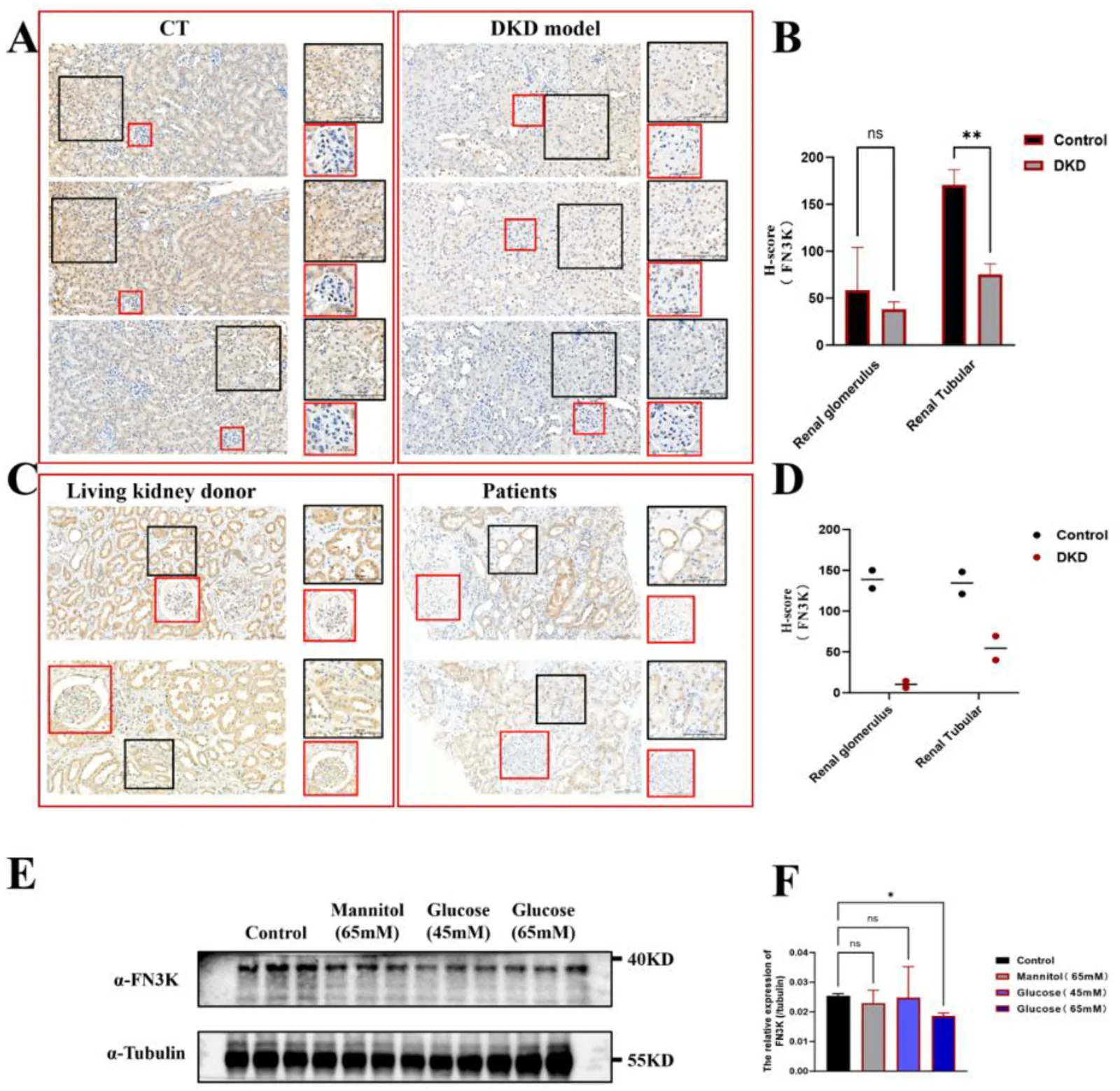
Reduced FN3K expression in renal tubular compartments in diabetic kidney disease. **(A)** Representative immunohistochemical (IHC) staining of FN3K in kidney sections from control (CT) and DKD model mice, with n = 3 independent biological replicates respectively. The red boxes indicate glomerular regions, and the black boxes indicate tubular regions. Corresponding higher-magnification insets are shown on the right. **(B)** Quantification of FN3K expression by H-score analysis in the glomerular and tubular compartments of control and DKD mice. FN3K expression was significantly reduced in the renal tubules but not in the glomeruli of DKD mice. **(C)** Representative FN3K IHC staining of kidney biopsy samples from living kidney donors and patients with diabetic kidney disease, with n = 2 independent biological replicates respectively. The black and red boxes denote the glomerular and tubular areas, respectively, with magnified views shown. **(D)** H-score quantification of FN3K expression in glomerular and tubular regions from human kidney samples, demonstrating marked tubular downregulation of FN3K in DKD patients. **(E)** Western blot analysis of FN3K protein levels in HK-2 cells treated with normal glucose, mannitol (65 mM), or high glucose (45 mM and 65 mM), with n = 3 independent biological replicates respectively. Tubulin was used as a loading control. **(F)** Densitometric quantification of FN3K protein expression normalized to that of tubulin. High-glucose treatment significantly reduced FN3K expression, whereas mannitol had no significant effect. The data are presented as the means ± SDs. ns, not significant; P values were determined via Student’s t test or one-way ANOVA as appropriate *, P values <0.05; **, P values<0.01.

Although FN3K has been reported to be enriched in the kidney, its expression pattern in DKD has not been clearly defined. To address this, DKD animal models were established via a high-fat diet combined with STZ administration. IHC analysis demonstrated that FN3K expression was significantly lower in the renal tubular compartments of DKD mice than in those of control mice, as reflected by a lower H-score (p = 0.003) (**Figures 1A, B**). In contrast, FN3K expression in the glomeruli did not differ significantly between the control and DKD groups (p = 0.579) (**Figures 1A, B**).

Consistently, kidney biopsy samples from patients with DKD presented markedly decreased FN3K expression within tubular regions, confirming the tubule-specific downregulation of FN3K in human DKD (**Figures 1C, D**). To determine whether FN3K downregulation is associated with hyperglycemia-induced tubular injury, human proximal tubular epithelial cells (HK-2) were exposed to high-glucose conditions. Compared with normal glucose conditions, treatment with 65 mM glucose significantly decreased FN3K protein levels (p = 0.021) (**Figures 1E, F**).

### 3.2 Serum FN3K levels are negatively associated with DKD-related clinical indices

To investigate the association between circulating FN3K levels and DKD progression, clinical data, including data from 46 individuals with nondiabetic kidney disease (NDKD) and 84 patients with DKD, were extracted from the KPMP cohort. The patients’ baseline characteristics are summarized in **Table 1**. No significant differences in sex or serum creatinine level were observed between the two groups.

Univariate logistic regression analysis revealed that serum FN3K (OR = 0.612, p = 0.017) and albumin (OR = 0.331, p = 0.004) were negatively associated with DKD risk, whereas blood urea nitrogen (OR = 1.136, p < 0.001), cystatin C (OR = 1.742, p = 0.010), HbA1c >7% (OR = 4.378, p < 0.001), and age (OR = 6.056, p < 0.001) were positively associated with DKD risk (**Table 2**).

**Table 2:** Univariate logistic regression analysis of clinical factors associated with diabetic kidney disease (DKD).

Clinical index	OR	95%CI	P
FN3K (Scale)	0.612	0.396-0.894	1.71E-02
Albumin (g/dL)	0.331	0.148-0.68	4.33E-03
Blood urea nitrogen (mg/dL)	1.136	1.084-1.201	1.00E-06
Creatinine (mg/dL)	1.198	0.884-1.876	3.36E-01
Cystatin C (mg/L)	1.742	1.164-2.713	9.98E-03
Urine Protein (mg/dL)	1.001	0.998-1.002	9.93E-01
HbA1c	4.378	1.975-10.405	4.49E-04
eGFR (ml/min/1.73m^2^)	0.999	0.998-1.003	9.93E-01
Age	6.056	2.958-14.846	7.00E-06

Odds ratios (ORs) and 95% confidence intervals (CIs) were estimated via univariate logistic regression models. The FN3K levels were standardized (scaled) prior to analysis. Continuous variables were analyzed per unit increase, and HbA1c was analyzed as a categorical variable. P values indicate the significance of associations with DKD risk.

Variables with statistical significance in univariate analysis, along with age and sex as potential confounders, were subsequently included in multivariate logistic regression models. FN3K remained significantly and negatively associated with DKD risk in the unadjusted model. This association persisted after sequential adjustment for age, sex, race, albumin, blood urea nitrogen, and HbA1c, indicating that FN3K was independently associated with reduced DKD risk (**Table 3**). When FN3K expression was categorized into tertiles, patients in the highest tertile (T3) and middle tertile (T2) presented a significantly increased risk of DKD compared with those in the lowest tertile (T1) across all the models (**Table 3**). In the fully adjusted multivariable model, age, sex, race, albumin, blood urea nitrogen, and HbA1c, FN3K expression, age, HbA1c, and BUN were identified as independent risk factors for DKD (**Table 4**).

**Table 3:** Multivariable logistic regression models evaluating the association between FN3K expression and diabetic kidney disease (DKD).

Project	Model1		Model2		Model3	
	OR(95%CI)	P	OR(95%CI)	P	OR(95%CI)	P
FN3K (per SD increase)	0.612(0.406-0.916)	0.017	0.610(0.389-0.958)	0.032	0.471(0.262-0.849)	0.012
FN3K intensity tertiles						
T1	Ref.		Ref.		Ref.	
T2	0.137(0.041-0.453)	0.001	0.084(0.021-0.335)	<0.0001	0.079(0.016-0.382)	0.002
T3	0.081(0.025-0.267)	<0.0001	0.061(0.015-0.242)	<0.0001	0.036(0.004-0.344)	0.004

Odds ratios (ORs) with 95% confidence intervals (CIs) are shown for three sequential logistic regression models. FN3K was analyzed both as a continuous variable (per standard deviation [SD] increase) and as tertiles (T1–T3), with the lowest tertile (T1) used as the reference. Model 1 included FN3K only; Model 2 was additionally adjusted for age and sex; Model 3 was further adjusted for race, albumin, blood urea nitrogen, and HbA1c. P values indicate two-sided tests.

**Table 4:** Multivariate logistic regression analysis of factors associated with diabetic kidney disease (DKD).

Clinical index	OR	95%CI	P
FN3K (per SD increase)	0.471	0.262-0.849	1.20E-02
Age	11.164	2.790-44.673	1.00E-03
Sex	1.929	0.650-5.724	2.37E-01
Race	3.108	0.112-86.599	5.04E-01
Albumin	0.838	0.298-2.351	7.37E-01
HbA1c	4.332	1.263-14.856	2.00E-02
Cystatin C	1.298	0.769-2.194	3.29E-01
Blood urea nitrogen	1.070	1.014-1.130	1.40E-02

Odds ratios (ORs) with 95% confidence intervals (CIs) were estimated via a multivariable logistic regression model including FN3K (per standard deviation [SD] increase), age, sex, race, albumin, HbA1c, cystatin C, and blood urea nitrogen. P values represent two-sided tests evaluating associations with the presence of DKD.

**Figure 2:**
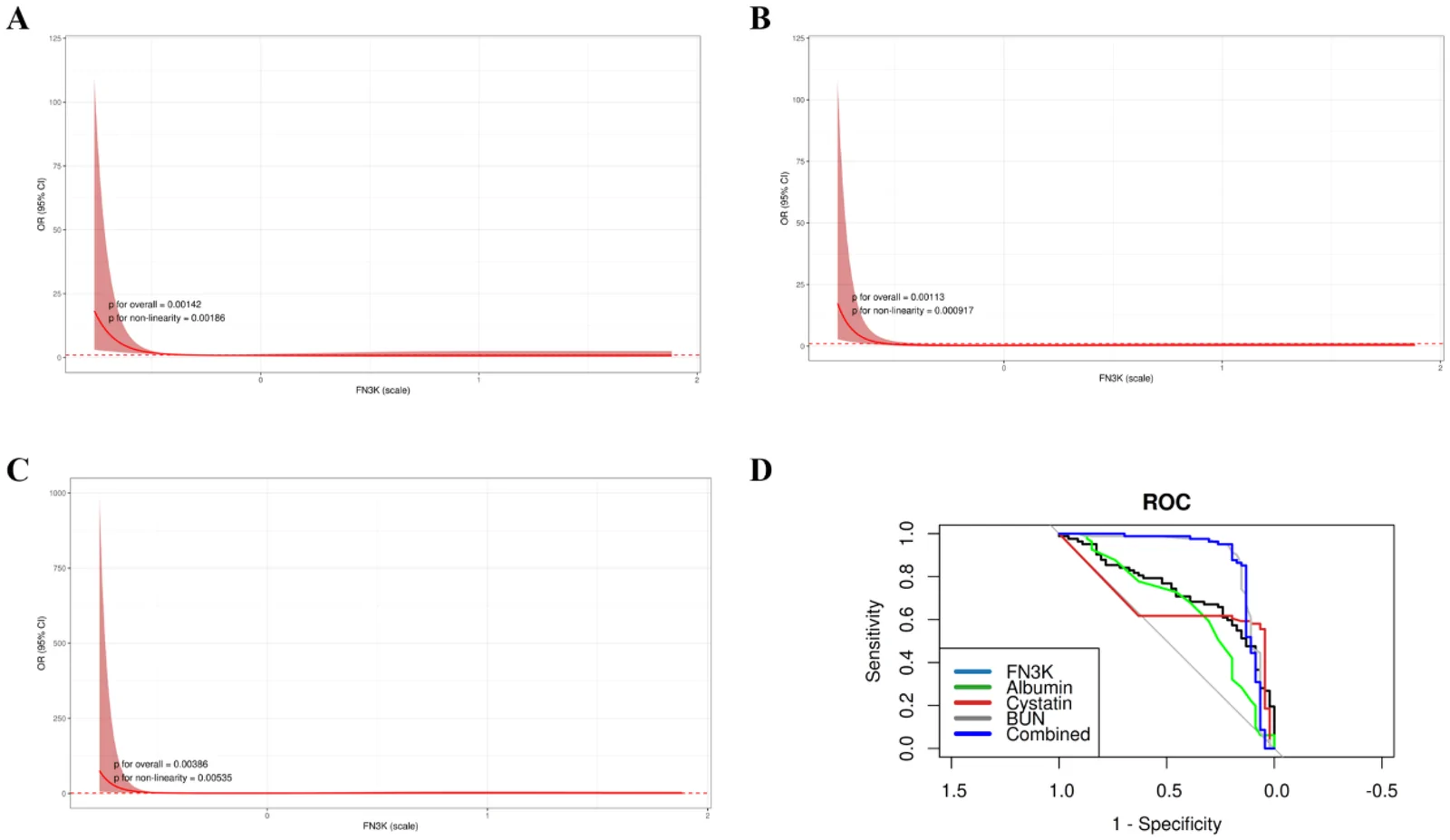
Nonlinear associations between serum FN3K levels and DKD risk and the diagnostic performance of FN3K. **(A–C)** Restricted cubic spline (RCS) analyses illustrating the nonlinear association between standardized serum FN3K levels and the odds of diabetic kidney disease (DKD). Odds ratios (ORs) and 95% confidence intervals are shown. Model 1 included FN3K only **(A)**; Model 2 was additionally adjusted for age and sex **(B)**; Model 3 was further adjusted for race, albumin, blood urea nitrogen, and HbA1c **(C)**. P values for overall association and nonlinearity are indicated in each panel. **(D)** Receiver operating characteristic (ROC) curve analysis evaluating the diagnostic performance of FN3K, albumin, cystatin C, and blood urea nitrogen (BUN) for DKD. Compared with the individual markers, the combined model incorporating FN3K, albumin, cystatin C, and BUN demonstrated improved diagnostic accuracy.

Restricted cubic spline regression analysis further demonstrated a nonlinear relationship between FN3K levels and DKD risk after adjustment for age, sex, race, albumin, blood urea nitrogen, and HbA1c (nonlinear p < 0.05) (**Figures 2A–C**).

Receiver operating characteristic (ROC) curve analysis was performed to evaluate the diagnostic performance of FN3K for DKD (**Figure 2D**). The optimal cut-off value of FN3K was −0.326, yielding a sensitivity of 65.9%, a specificity of 76.1%, and an area under the curve (AUC) of 0.762 (95% CI: 0.639–0.814, p < 0.001). The optimal cut-off value of the serum ALB concentration for diagnosing DKD was 4.05, with a sensitivity of 59.3% and a specificity of 67.0%, and the area under the curve (AUC) was 0.660 (95% CI: 0.559–0.760, P<0.001). The optimal cut-off value of cystatin C for diagnosing DKD was 4.515, with a sensitivity of 100% and a specificity of 2.2%, and the area under the curve (AUC) was 0.34 (95% CI: 0.246–0.434, P<0.001). The optimal cut-off value of BUN for diagnosing DKD was 70.0, with a sensitivity of 100% and a specificity of 2.1%, and the area under the curve (AUC) was 0.119 (95% CI: 0.041–0.198, P<0.001). The AUC for diagnosing DKD via the FN3K index combined with albumin, cystatin C, and BUN was 0.880 (95% CI: 0.800–0.960, P<0.001), with a sensitivity of 95.1% and a specificity of 80.4%.

### 3.3 Regional proteomic analysis reveals the activation of PI3K/AKT signaling and the suppression of mitochondrial pathways in DKD tubules

**Figure 3:**
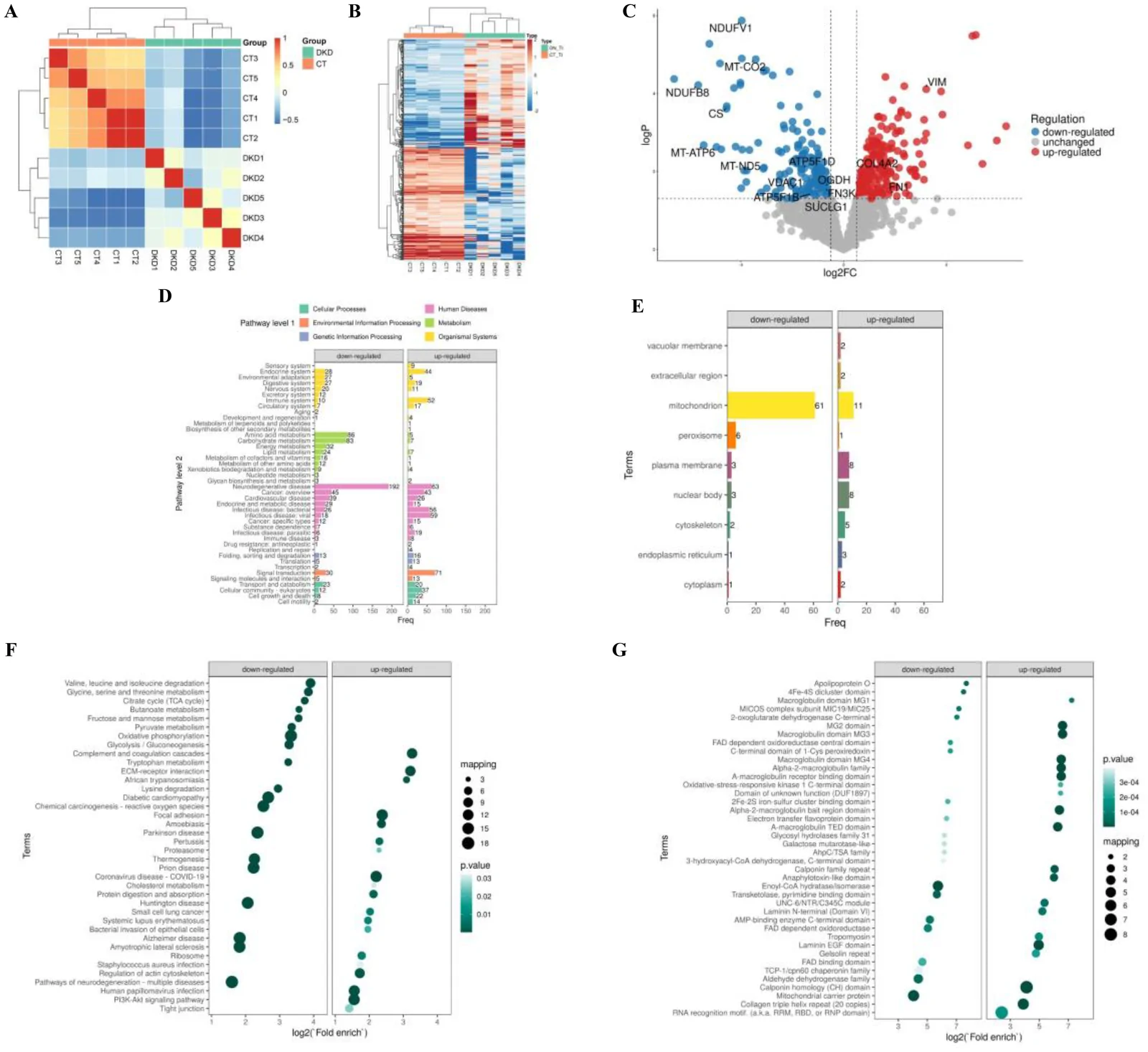
Regional proteomic profiling reveals mitochondrial suppression and activation of PI3K/AKT signaling in DKD tubules. **(A)** Heatmap showing unsupervised hierarchical clustering of differentially expressed proteins in tubulointerstitial samples from control (CT) and diabetic kidney disease (DKD) kidneys. **(B)** Heatmap illustrating the relative expression patterns of differentially expressed proteins between CT and DKD samples. **(C)** Volcano plot displaying differentially expressed proteins in DKD tubules compared with controls. Downregulated proteins are shown in blue, and upregulated proteins are shown in red. Key mitochondrial proteins and injury-associated markers are highlighted. **(D)** Kyoto Encyclopedia of Genes and Genomes (KEGG) pathway classification of differentially expressed proteins, stratified by upregulated and downregulated pathways. **(E)** Subcellular localization analysis of the differentially expressed proteins predicted by WoLF PSORT, which revealed a marked reduction in the number of mitochondrion-localized proteins in DKD tubules. **(F)** KEGG pathway enrichment analysis of downregulated and upregulated proteins. **(G)** Protein domain enrichment analysis of differentially expressed proteins. The dot size represents the number of mapped proteins, and the color intensity indicates statistical significance. Enrichment analyses were performed via Fisher’s exact test, with P < 0.05 considered statistically significant.

To further investigate the molecular alterations associated with DKD-related tubular injury, regional proteomic analysis was performed on tubulointerstitial samples obtained from five kidney stone donors and five patients with DKD. A total of 1,473 proteins were identified, among which 331 proteins were differentially expressed (|log2-fold change| > 1, p < 0.05) (**Figures 3A, B**).

Among the proteins upregulated in DKD tubules were markers associated with tubular injury and extracellular matrix remodeling, including vimentin (VIM; 3.216-fold), fibronectin 1 (FN1; 1.743-fold), and collagen type IV alpha 2 chain (COL4A2; 1.721-fold). Moreover, FN3K was significantly downregulated in DKD tubules. In addition, multiple proteins involved in mitochondrial function were markedly reduced, including NADH:ubiquinone oxidoreductase subunit B8 (NDUFB8; 5.669-fold decrease), citrate synthase (CS; 4.557-fold decrease), mitochondrially encoded ATP synthase membrane subunit 6 (MT-ATP6; 5.455-fold decrease), mitochondrially encoded NADH dehydrogenase subunit 5 (MT-ND5; 3.105-fold decrease), mitochondrially encoded cytochrome c oxidase subunit II (MT-CO2; 3.426-fold decrease), ATP synthase F1 subunit delta (ATP5F1D; 1.646-fold decrease), voltage-dependent anion channel 1 (VDAC1; 1.855-fold decrease), oxoglutarate dehydrogenase (OGDH; 0.783-fold decrease), and succinyl-CoA ligase GDP-forming subunit alpha (SUCLG1; 1.090-fold decrease) (**Figure 3C**).

Functional classification analysis revealed that the number of differentially expressed proteins involved in carbohydrate metabolism, amino acid metabolism, energy metabolism, and lipid metabolism was reduced in DKD samples (**Figure 3D**). In contrast, proteins related to immune system processes were increased, indicating enhanced immune activation in DKD tubules (**Figure 3D**). Subcellular localization analysis via WoLF PSORT demonstrated a marked reduction in the number of mitochondrion-localized proteins among the differentially expressed proteins in DKD patients (**Figure 3E**).

KEGG pathway enrichment analysis revealed significant downregulation of mitochondrial-related pathways, including the tricarboxylic acid (TCA) cycle and oxidative phosphorylation, whereas complement and coagulation cascades and the PI3K/AKT signaling pathway were significantly upregulated (**Figure 3F**).

Protein domain enrichment analysis further revealed coordinated alterations in domains related to extracellular matrix organization, immune and complement activation, protease inhibition, and mitochondrial metabolism (**Figure 3G**).

### 3.4 Single-cell RNA sequencing links FN3K expression to mitochondrial function in tubular cells

**Figure 4:**
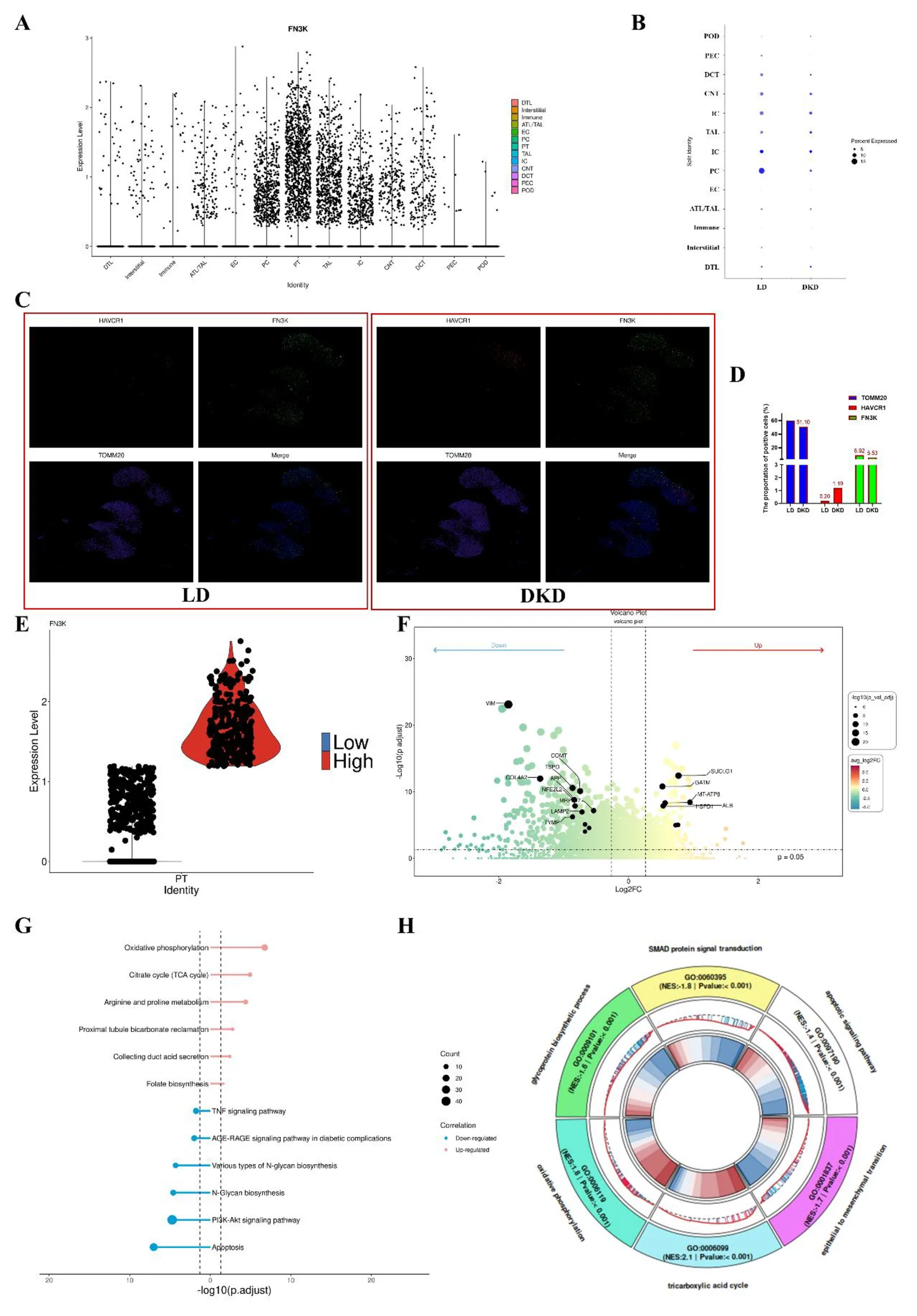
Single-cell transcriptomic analysis links FN3K expression to tubular injury, mitochondrial function, and PI3K/AKT signaling. **(A)** Violin plot showing FN3K expression across major renal cell populations identified by single-cell RNA sequencing, including PTs, distal tubular segments, and other renal cell types. **(B)** Dot plot illustrating FN3K expression patterns across renal cell types in living donor (LD) and DKD samples. The dot size represents the proportion of cells expressing FN3K, and the color intensity indicates the average expression level. **(C)** scRNA images showing costaining of FN3K, HAVCR1, and the mitochondrial marker TOMM20 in kidney tissues from living donors (LDs) and DKD patients. **(D)** Quantification of the proportions of TOMM20-, HAVCR1-, and FN3K-positive cells in LD and DKD samples, confirming that mitochondrial loss and tubular injury are accompanied by reduced FN3K expression in DKD. **(E)** Violin plot showing stratification of PT cells into FN3K-high and FN3K-low groups on the basis of the FN3K expression level. **(F)** Volcano plot depicting DEGs between FN3K-high and FN3K-low PT cells. Genes associated with tubular injury and fibrosis were upregulated in the FN3K-low group, whereas mitochondrial and metabolic genes were enriched in the FN3K-high group. **(G)** Gene set enrichment analysis (GSEA) of differentially expressed genes in PT cells. FN3K-high cells are enriched in mitochondrial metabolic pathways, including oxidative phosphorylation and the tricarboxylic acid (TCA) cycle, whereas FN3K-low cells are enriched in PI3K/AKT signaling, TNF signaling, and apoptosis-related pathways. **(H)** Circular GSEA visualization highlighting the coordinated regulation of key signaling and metabolic pathways associated with FN3K expression, including the TCA cycle and SMAD protein signaling. Dot size reflects gene count, and color indicates the direction and significance of enrichment. Statistical significance was assessed via adjusted P values, with thresholds as described in the Methods.

Single-cell RNA sequencing analysis revealed that FN3K was expressed across multiple tubular cell types, including proximal tubules (PTs), principal cells (PCs), connecting tubules (CNTs), thick ascending limbs (TALs), and distal convoluted tubules (DCTs). Compared with that in other tubular segments, FN3K expression was relatively greater in PTs and PCs (**Figures 4A, B**). Moreover, the proportions of FN3K- and TOMM20-positive cells were reduced in DKD-associated tubular cell populations, whereas the proportion of HAVCR1-positive cells was increased (**Figures 4C, D**).

To explore potential FN3K-associated transcriptional programs, PT cells were stratified into high- and low-FN3K expression groups (**Figure 4E**). Differential expression analysis revealed 2,314 genes that were significantly differentially expressed between the two groups (|log2-fold change| > 0.5, adjusted p < 0.05) (**Figure 4F**). Notably, the expression of tubular injury–associated genes such as VIM and COL4A2 was significantly downregulated in the FN3K-high group.

Gene set enrichment analysis revealed the upregulation of mitochondrial metabolic pathways, including the TCA cycle and oxidative phosphorylation, in the FN3K-high PT cells (**Figures 4G, H**). In contrast, the PI3K/AKT signaling, TNF signaling, and apoptosis-related pathways were significantly downregulated (**Figures 4G, H**).

### 3.5 FN3K-associated remodeling of mitochondrial metabolism

**Figure 5:**
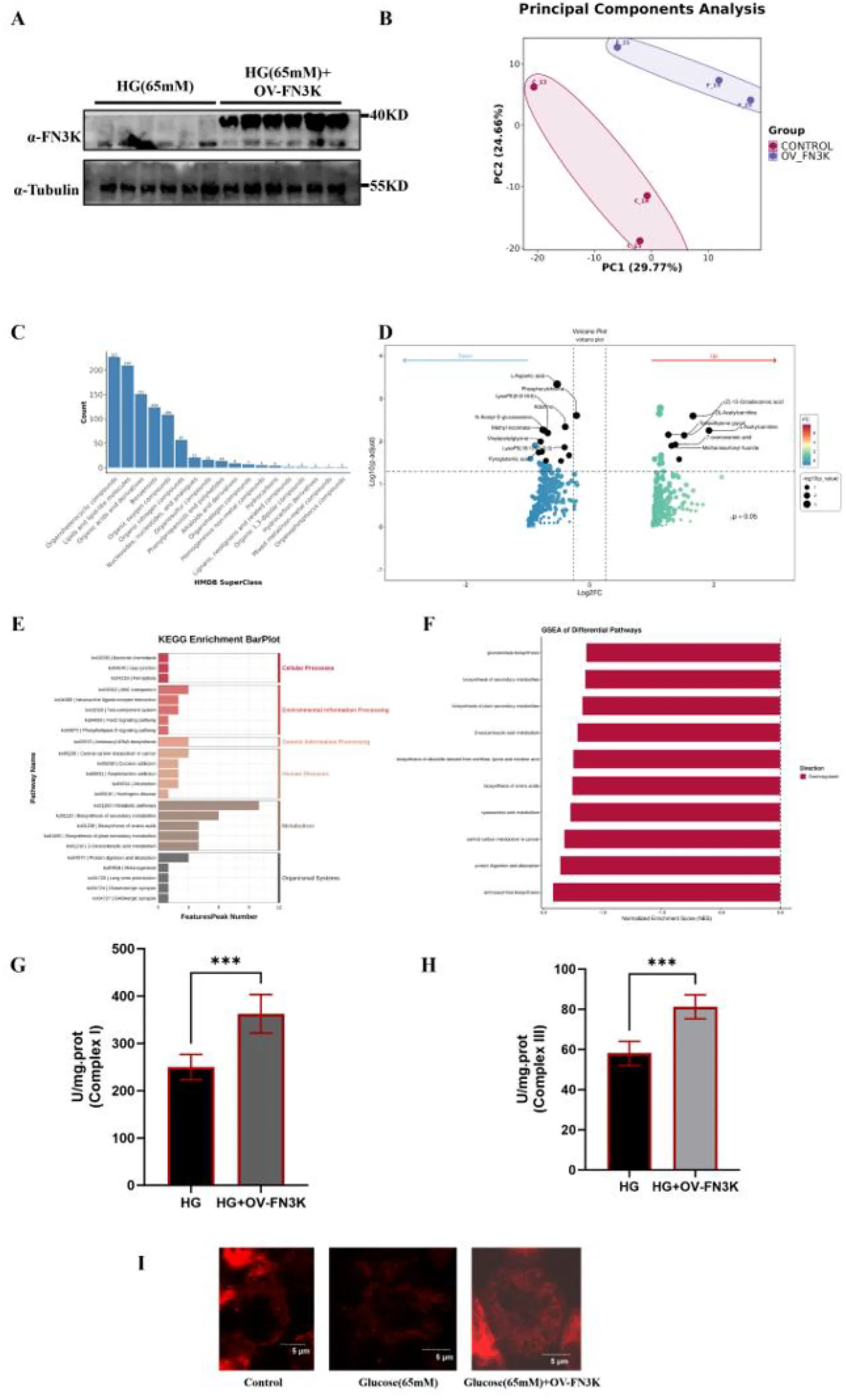
FN3K enhances mitochondrial function under high-glucose conditions. **(A)** Western blot analysis confirming FN3K overexpression in HK-2 cells cultured under high-glucose conditions (65 mM), with n = 6 independent biological replicates respectively. Tubulin served as the loading control. **(B)** Principal component analysis (PCA) score plot showing distinct separation between high glucose-treated control cells and FN3K-overexpressing cells, with ellipses indicating group clustering, with n = 3 independent biological replicates respectively. **(C)** Classification of identified metabolites on the basis of the Human Metabolome Database (HMDB) superclass, showing broad coverage of lipid, amino acid, and organic acid metabolites. **(D)** Volcano plot depicting differentially abundant metabolites between high-glucose control and FN3K-overexpressing cells. Upregulated and downregulated metabolites are highlighted, with representative metabolites annotated. **(E)** Kyoto Encyclopedia of Genes and Genomes (KEGG) pathway enrichment analysis of differentially abundant metabolites, indicating significant alterations in metabolic pathways associated with FN3K overexpression. **(F)** Gene set enrichment analysis (GSEA) of metabolic pathways revealed the coordinated downregulation of pathways related to central carbon and amino acid metabolism following FN3K overexpression. The data were analyzed via untargeted metabolomics, and statistically significant thresholds are described in the Methods section. **(G, H)** Functional assays further demonstrated that FN3K overexpression significantly enhanced the activities of mitochondrial respiratory chain complex I and complex III, with n = 3 independent biological replicates respectively. **(I)** Representative fluorescence images of HK-2 cells labeled with MitoTracker Red CMXRos under normal glucose (NG, 5.5 mM), high glucose (HG, 65 mM), HG + FN3K overexpression ***P values < 0.001.

To assess whether FN3K modulates mitochondrial metabolism, HK-2 cells were transfected with an FN3K expression plasmid, followed by untargeted metabolomic analysis via liquid chromatography–mass spectrometry (LC-MS). The transfection efficiency was validated via western blot analysis (**Figure 5A**). PCA revealed distinct metabolic profiles and high reproducibility. Score plots revealed tight clustering of biological replicates within each treatment group (HG, HG plus overexpression of FN3K), indicating a consistent metabolite composition (**Figure 5B**). After quality control, 613 metabolites, including lipids, lipid-like molecules, nucleotides, and intermediates involved in 2-oxocarboxylic acid metabolism, were identified (**Figure 5C**).

Differentially abundant metabolite analysis revealed 31 significantly altered metabolites (p < 0.05). Among these, the levels of mitochondria-related metabolites-including L-acetylcarnitine, DL-acetylcarnitine, and N-acetyl-L-glutamate (NAG)-were significantly increased. In contrast, metabolites associated with membrane lipid remodeling and inflammatory lipid signaling, such as LysoPE (0:0/18:0), LysoPE (P-16:0/0:0), LysoPE (P-18:1(9Z)/0:0), LysoPS (18:0/0:0), and LysoPS (18:1(9Z)/0:0), were downregulated. Adenine, previously reported as a risk-associated metabolite in DKD, was also significantly reduced following FN3K overexpression (**Figure 5D**).

KEGG enrichment analysis revealed significant enrichment of pathways related to arginine biosynthesis, central carbon metabolism, amino acid biosynthesis, 2-oxocarboxylic acid metabolism, and aminoacyl-tRNA biosynthesis (**Figure 5E**). Metabolite set enrichment analysis further indicated the downregulation of aminoacyl-tRNA biosynthesis and central carbon metabolism (**Figure 5F**).

To validate whether FN3K is associated with mitochondrial function, we transferred FN3K to HK2 cells and estimated the activities of mitochondrial respiratory chain complex I and complex III. Functional assays further demonstrated that FN3K overexpression significantly enhanced the activities of mitochondrial respiratory chain complex I (**Figure 5G**) and complex III (**Figure 5H**), suggesting improved mitochondrial oxidative phosphorylation capacity. In addition, FN3K preserves mitochondrial numbers in HK-2 cells under high-glucose conditions (**Figure 5I**).

### 3.6 Integrated pathway analysis identifies shared metabolic signatures across transcriptomic and metabolomic datasets

**Figure 6:**
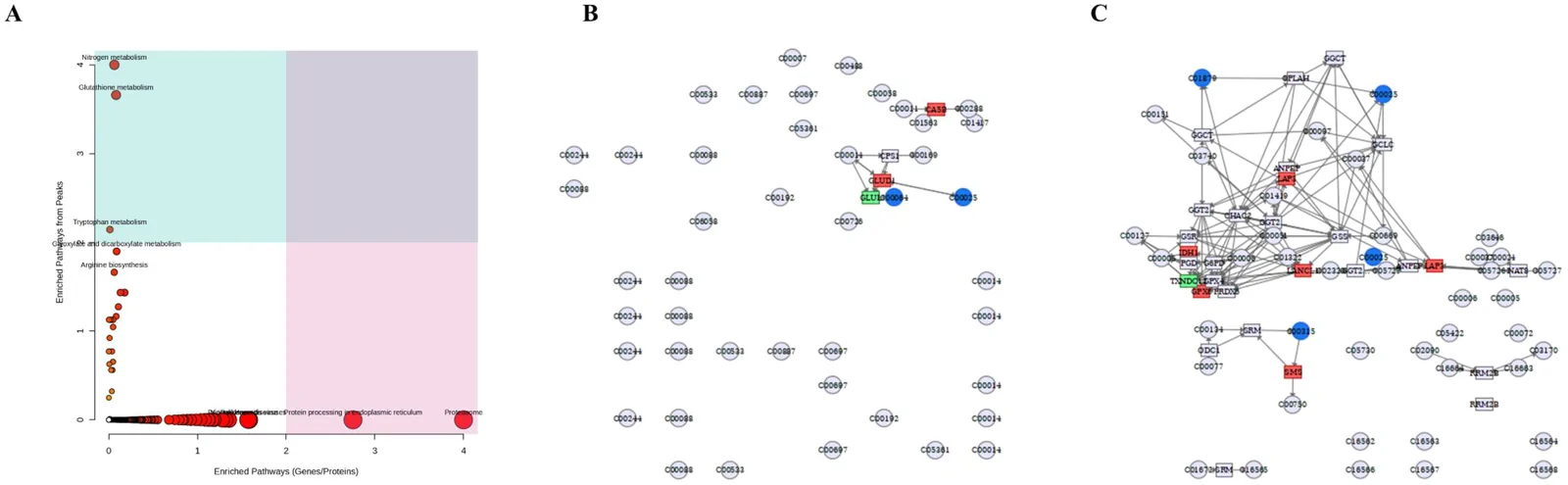
Integrated transcriptomic and metabolomic analyses identified nitrogen and glutathione metabolism as shared FN3K-associated pathways. **(A)** Joint pathway analysis integrating single-cell transcriptomic and untargeted metabolomic data, highlighting significantly enriched pathways at both the gene and metabolite levels. Nitrogen metabolism and glutathione metabolism were among the top pathways associated with FN3K expression. **(B)** Metabolite–gene interaction network for the nitrogen metabolism pathway, showing relationships between differentially abundant metabolites and differentially expressed genes mapped to the same KEGG pathway. **(C)** Metabolite–gene interaction network for the glutathione metabolism pathway, illustrating coordinated alterations in metabolites and genes involved in redox regulation and cellular stress responses. Nodes represent genes or metabolites, and edges indicate known or predicted interactions. Network construction and enrichment analyses were performed via MetaboAnalyst and Cytoscape, as described in the Methods section.

To integrate the transcriptomic and metabolomic findings, joint pathway analysis was performed via MetaboAnalyst. Pathways such as the TCA cycle, apoptosis, and oxidative phosphorylation were enriched primarily at the gene expression level, whereas aminoacyl-tRNA biosynthesis was enriched predominantly at the metabolite level. Notably, nitrogen metabolism and glutathione metabolism were enriched in both datasets (**Figure 6A**).

Within the nitrogen metabolism pathway, the key metabolites included L-glutamate and L-glutamine, whereas the associated genes included carbonic anhydrase 5B (CA5B), glutamate dehydrogenase 1 (GLUD1), and glutamate-ammonia ligase (GLUL) (**Figure 6B**). The glutathione metabolism pathway comprises metabolites such as L-glutamate, 5-oxoproline, and spermidine, along with multiple proteasome-related genes (**Figure 6C**).

## 4 Discussion

Diabetic kidney disease (DKD) is one of the most severe microvascular complications of diabetes and remains a leading cause of end-stage kidney disease (ESKD), affecting nearly 40% of individuals with diabetes (35, 36). Although DKD has traditionally been regarded as a glomerulus-centered disease, accumulating evidence indicates that tubular injury plays a pivotal role in its early onset and progression. Tubulointerstitial fibrosis, epithelial dedifferentiation, and apoptosis can occur at early stages of diabetes, in some cases preceding the development of overt proteinuria (37–40). Accordingly, several tubular injury-associated biomarkers, such as kidney injury molecule-1 (KIM-1) and neutrophil gelatinase–associated lipocalin (NGAL), have been proposed for DKD assessment (41). However, their ability to independently predict disease progression remains limited, suggesting that markers reflecting earlier pathogenic events may be needed. In this context, our study demonstrated that FN3K expression was markedly reduced in the tubular compartments of DKD patients but remained largely unchanged in the glomerulus, supporting the tubule-specific involvement of FN3K in DKD pathogenesis. This compartment-specific reduction is biologically plausible, as renal tubular epithelial cells are exposed to exceptionally high metabolic demand, glucose overload, and sustained glycation stress under diabetic conditions, rendering them particularly vulnerable to disturbances in protein quality control and repair mechanisms. FN3K is a key enzyme involved in the removal of early glycation adducts, and previous studies have shown that reduced FN3K levels in erythrocytes are associated with a positive glycation gap (GGap), a phenotype reflecting disproportionate glycation relative to circulating glycemic exposure and impaired glycation repair capacity in severe diabetes. Extending these observations to DKD, our data further revealed that serum FN3K was negatively correlated with the risk of DKD and remained an independent protective factor after multivariable adjustment. Furthermore, ROC analysis demonstrated that a combined model incorporating FN3K, albumin, cystatin C, and BUN levels achieved superior diagnostic performance for DKD than individual biomarkers did. Collectively, these findings suggest that reduced FN3K expression may increase tubular susceptibility by compromising glycation repair capacity, thereby promoting tubular dysfunction and accelerating DKD progression. Unlike conventional tubular injury markers that primarily reflect downstream epithelial damage, FN3K may capture upstream metabolic vulnerability, providing complementary insight into the pathogenesis of DKD.

Fructosamine-3-kinase (FN3K) is a key enzyme involved in the repair of early nonenzymatic glycation adducts through the phosphorylation of fructosamine-lysine residues, thereby facilitating their degradation and limiting the accumulation of advanced glycation end products (AGEs) (16, 42). By restraining glycation stress, FN3K has been recognized as an important regulator of protein quality control under hyperglycemic conditions (43, 44). Previous studies have demonstrated that FN3K gene polymorphisms are closely associated with diabetes-related clinical indices, including HbA1c levels (14). Notably, the rs1056534 polymorphism in FN3K has been linked to elevated HbA1c levels and an increased risk of type 2 diabetes, suggesting that FN3K-mediated glycation repair may influence long-term diabetic outcomes (45). In addition to systemic glycemic markers, accumulating evidence supports a pathogenic role for AGEs in diabetic nephropathy. Near-infrared spectroscopy analyses of renal tissue from patients with DKD have revealed significant correlations between AGE-related metabolites and impaired renal function, highlighting AGEs as key mediators of renal injury (6). In this context, impaired FN3K activity may exacerbate downstream proinflammatory and proapoptotic signaling in the diabetic kidney. Mechanistically, our results demonstrated that increased FN3K expression in PT cells was associated with reduced apoptosis, attenuated TNF signaling, and suppression of the AGE-RAGE pathway, accompanied by increased TCA cycle activity and oxidative phosphorylation. Notably, a previous study suggested that FN3K is translated into mitochondria and promotes protein translation, which would alleviate mitochondrial function (46). Moreover, FN3K is also involved in antioxidant and cell stress programs via the deglycation of nuclear factor-erythroid 2 related factor 2 (Nrf2) in liver cancer (47). 394.

Mitochondria serve as the central hub of cellular bioenergetics and are particularly critical for renal tubular cells, which rely heavily on oxidative metabolism to sustain solute transport and reabsorption (48–50). Disruption of mitochondrial quality control, dynamics, or metabolism leads to excessive reactive oxygen species (ROS) production and oxidative stress, thereby promoting tubular injury (23). Under sustained hyperglycemic conditions, mitochondrial dysfunction in renal tubules is characterized by reduced ATP generation and impaired oxidative phosphorylation, findings that are consistent with our regional tubular proteomic data. In parallel, we observed upregulation of the PI3K/AKT signaling pathway in DKD tubules. Previous studies have reported that high glucose can induce aberrant activation of PI3K/AKT signaling, which in turn suppresses mitophagy through mTOR activation and exacerbates tubular apoptosis (51–53). Notably, our single-cell RNA-sequencing analyses revealed that increased FN3K expression in PT cells was associated with decreased PI3K/AKT pathway activity, reduced apoptotic signaling, and increased oxidative phosphorylation and TCA cycle activity, suggesting an inverse association between FN3K expression and maladaptive PI3K/AKT activation. While these findings do not establish a direct regulatory interaction, they support the notion that preservation of FN3K expression is linked to improved mitochondrial homeostasis and cellular resilience under diabetic stress. In addition, our metabolomic analyses revealed significant upregulation of mitochondrial function-related metabolites, including L-acetylcarnitine, DL-acetylcarnitine, and N-acetyl-L-glutamate. The accumulation of acetylcarnitine reflects enhanced buffering of acetyl-CoA and preservation of free CoA availability, thereby supporting sustained TCA cycle flux and oxidative phosphorylation (54). Concurrently, elevated N-acetyl-L-glutamate suggests the activation of mitochondrial nitrogen metabolism, facilitating metabolic flexibility in response to diabetic stress (55). Together, these metabolic adaptations further support a protective role for FN3K in maintaining mitochondrial function in diabetic tubules. Collectively, our findings support a model in which FN3K deficiency contributes to early tubular vulnerability in DKD by amplifying glycation stress, disrupting mitochondrial metabolism, and promoting maladaptive PI3K/AKT signaling.

In addition, when transcriptomic and metabolomic findings are integrated, nitrogen metabolism and glutathione metabolism, which are tightly integrated with mitochondrial function, are particularly critical for maintaining the physiological integrity of renal tubular cells. are enriched in both datasets. Nitrogen metabolism refers to the collective biochemical processes responsible for the synthesis, interconversion, and degradation of nitrogen-containing compounds, primarily amino acids, ammonia, urea, and related metabolites (56). In mammalian cells, nitrogen metabolism is inseparably linked to amino acid turnover and energy metabolism, with mitochondria serving as the central hub for these processes (57). GLUD1 catalyzes the reversible conversion of glutamate to α-ketoglutarate with the release of free ammonia. This reaction directly links nitrogen flux to the TCA cycle, allowing nitrogen metabolism to support mitochondrial oxidative phosphorylation (58). Our results showed that the overexpression of FN3K promoted the expression of GLUD1. Moreover, glutathione metabolism plays a central role in cellular redox regulation and mitochondrial homeostasis and is critically involved in the pathogenesis of metabolic and kidney diseases (59). Glutathione (GSH), the most abundant intracellular nonprotein thiol, serves as a major antioxidant by directly scavenging reactive oxygen species (ROS) and acting as an essential cofactor for glutathione peroxidases (60). GPX6 and IDH1 are key components of the cellular antioxidant network, contributing to ROS detoxification and redox homeostasis through glutathione-dependent and NADPH-generating pathways, respectively (61, 62). Given the established role of FN3K in limiting protein glycation and glycation-induced oxidative stress, our findings suggest that FN3K overexpression mitigates mitochondrial metabolic dysfunction by reducing the oxidative burden and increasing the antioxidant defense capacity.

However, our study has several limitations. First, although our multiomics analyses revealed significant associations between FN3K expression, glutathione and nitrogen metabolism, and mitochondrial dysfunction, the findings of the current study are largely correlative and do not establish direct causal relationships. Second, mechanistic validation was primarily performed *in vitro* using HK-2 cells under high-glucose conditions, which may not fully recapitulate the complex metabolic and cellular interactions present in diabetic kidney disease *in vivo*. Third, although human kidney biopsy samples were included to support the clinical relevance of our findings, the sample size was relatively limited, and detailed longitudinal data were not available. Forth, while our multi-omics analyses revealed an association between FN3K expression and altered PI3K/AKT signaling in DKD tubules, we did not perform direct pharmacological interrogation of this pathway to establish causality. Given the well-documented context-dependence of PI3K/AKT signaling, dissecting its precise mechanistic relationship with FN3K-mediated mitochondrial protection will require dedicated future investigation. In addition, direct assessment of mitochondrial function, such as measurements of respiratory capacity or mitochondrial redox status, has not been systematically performed. Future studies incorporating genetic models, longitudinal patient cohorts, and comprehensive functional assays will be needed to further delineate the causal role of FN3K in mitochondrial metabolism and diabetic tubular injury.

## 5 Conclusions

FN3K deficiency appears to be closely associated with the development and progression of diabetic kidney disease (DKD). Reduced FN3K expression may lead to the accumulation of early glycation products and increased glycation stress, thereby exacerbating cellular damage in renal tubular epithelial cells. This glycation imbalance is likely accompanied by mitochondrial metabolic dysfunction, impaired energy production and increased oxidative stress.

## Data Availability

The original contributions presented in the study are included in the article/supplementary material. Further inquiries can be directed to the corresponding author.
